# Forensic application of phylogenetic analysis – exploration of suspected epidemiological linkage

**DOI:** 10.1186/1471-2334-14-S4-O21

**Published:** 2014-05-29

**Authors:** Marina Siljic, Dubravka Salemovic, Djordje Jevtovic, Ivana Pesic-Pavlovic, Sonja Zerjav, Valentina Nikolic, Jovan Ranin, Maja Stanojevic

**Affiliations:** 1 Institute of Microbiology and Immunology, University of Belgrade Faculty of Medicine, Belgrade, Serbia; 2 HIV/AIDS Unit, Institute for Infectious and Tropical Diseases CCS, Belgrade, Serbia; 3 Virology Department, Clinical Center Serbia, Belgrade, Serbia

## 

Phylogenetic analysis may serve as a valuable tool in assessing the epidemiological relation between viral DNA sequences. In order to increase the likelihood of observing phylogenetic separation of sequences as well as to give strong forensic evidence regarding transmission, phylogenetic analysis needs to be performed on appropriate local control sequences and by sequencing of at least two genetic regions of reasonable length, depending on the gene under investigation. The aim of this study was to explore the suspected epidemiological linkage between DNA sequences isolated from three HIV-1 infected patients by means of phylogenetic analyses.

Phylogenetic analysis was performed on investigated sequences together with a number of local controls, that are viral sequences from infected individuals in the same location and diagnosed in a similar time period. Two genetic regions, of 1,6 kb for pol and 800 bp for env, were amplified and sequenced from viral RNA extracted from plasma. Transmission clusters were assigned as those phylogenetic clades consisting of three or more sequences, fulfilling the conditions of genetic distance of 1.5% or less with posterior probability higher than 0.9 in the Bayesian analyses, for both genetic region. Maximum likelihood (ML) phylogenetic analysis was performed as implemented in Phylogenetic Analysis Using Parsimony (PAUP), using the evolutionary model selected by jModeltest software.

Phylogenetic analysis revealed the presence of a single transmission cluster that accomplished all predefined sets of criteria. This cluster was composed of three viral sequences isolated from patients whose epidemiological linkage was under investigation. The mean pairwise nucleotide divergence among all observed pol sequences was 7.1% (range 0.2-12.8%) while among sequences under investigation it was 0.8% (0.3-1.1%). Regarding env sequences, nucleotide divergence among all was 16.1% (range 0.3-26.3%), while among sequences under investigation it was 1.3% (1.1-1.5%).

Our results strongly support the epidemiological linkage between sequences under investigation. Viral phylogenies can help to evaluate proposed epidemiological linkage and to infer the ancestral relationships of infections. However phylogenetic analysis cannot unambiguously prove that HIV-1 transmission occurred directly between two individuals, since any transmission chain may contain additional sequences not included in the analysis.

